# Utilizing electronic resources to promote your residency program

**DOI:** 10.4322/acr.2021.286

**Published:** 2021-06-04

**Authors:** Colton Mabis, Momal Tara Chand, Shelby Miller

**Affiliations:** 1 St. George’s University, School of Medicine, Detroit, Mi, USA; 2 Ascension St John Hospital, Department of Pathology, Detroit, Mi, USA; 3 Ascension St John Hospital, Department of Research, Detroit, Mi, USA

**Keywords:** Coronavirus Infections, Internship and Residency, Pandemics

## Abstract

As the COVID-19 pandemic spread to the United States, it was followed by unprecedented changes. These changes did not spare undergraduate and graduate medical students. Specifically, medical students applying for residency programs were faced with a novel challenge. In March 2020, as the pandemic became increasingly severe, the Association of American Medical Colleges (AAMC) recommended pulling medical students from in-person clinical rotations. By May 2020, the AAMC recommended that all residency interviews be conducted online for the 2020-2021 residency application cycle. These unprecedented modifications to the interview season required programs to quickly adapt and find ways to utilize online tools to convey what their program offered to applicants. In this paper, we will outline the adaptations, tools, and resources that residencies and applicants have used to navigate through the 2020/2021 interview cycle.

Dear Editor

As the COVID-19 pandemic spread to the United States, it was followed by unprecedented changes. These changes did not spare undergraduate and graduate medical education. In March 2020,^1^ as the pandemic became increasingly severe, the Association of American Medical Colleges (AAMC) recommended pulling medical students from in-person clinical rotations. By May 2020,^2^ the AAMC recommended that all residency interviews be conducted online for the 2020-2021 residency application cycle. These unprecedented modifications to the interview season required programs to quickly adapt and find ways to utilize online tools to convey what their program offered to applicants. Programs utilized multiple virtual modalities, including their webpages, social media accounts, and teleconferencing. As the current interview season comes to a close, we look back on this past season to highlight best practices in residency programs’ online presence.

Although the pandemic accelerated the need for electronic residency program resources targeted to applicants, many of these changes will likely remain post-pandemic. It is probable that many programs will return to in-person interviews; however, research has shown that prospective residents are increasingly in favor of utilizing online resources to learn about residency programs. A 2014 study^3^ of residency program social media usage by applicants found that 47% of respondents had accessed the studied program’s Facebook page, and that it universally made them more likely to apply, interview, and rank the program. Regarding program web pages, a 2007 study^4^ found that, after geographic location, a quality program website had the most positive impact on an applicant's decision to apply to a particular program.

Traditionally, the residency interview season has involved medical students traveling to programs across the country, often completing sub-internships, attending pre-interview dinners with residents, hospital tours, and in-person interviews. These experiences allowed applicants to learn a great deal about the program, and importantly, to get a feel for the culture. A 2012 study found that “many of the factors that were ‘very important’ (to applicants) are not tangible but, instead, speak to the culture of an institution.” and that “Applicants appear to care most about collegiality and potential relationships with other residents and faculty.”^5^

Different electronic resources can effectively communicate different qualities of a program. Webpages offer an excellent mode to display the program curriculum, faculty, achievements, research, education, application requirements, and more. Social media has different advantages. Its dynamic and interactive nature is helpful in communicating residency culture. Teleconferencing offers prospective applicants the chance to speak directly with residents and faculty to further identify culture and have individual questions answered.

A crucial component of building a package of electronic resources is to create a unified brand identity. Programs that successfully engage in brand identification and translate it into an integrated online presence will have an easier time differentiating themselves from other programs. As discussed in a 2018 paper by Shappell et al.,^6^ “Brand identity is a construct that incorporates an organization's mission, vision, and values define a program, differentiates it from others in the specialty, and makes it relevant to specific target groups”. Further, identifying a program brand has significant overlap with the process of self-study outlined by the ACGME, and a “strong brand can shape culture, unify efforts, and align internal and external stakeholders.”

Program websites are one of the most universally used modes of an online presence. However, a 2018 study by Hansberry et al., analyzed 179 radiology program websites and found that very few offered a comprehensive set of content.^7^ Similarly, another 2018 study by Chen et al.,^8^ found that program websites “commonly include pertinent information that is useful; however, most are lacking components valued by applicants”. Previous studies have shown that candidates rely heavily on program websites when deciding where to apply, interview, and rank, thus making webpages more comprehensive would likely bolster recruitment efforts.

Social media is another valuable resource in the branding and recruitment toolbox and offers unique advantages over a webpage. Because social media is more dynamic than a webpage, it provides a medium for providing frequent updates, more personalized interactions, and a window into the daily life of residents. This collectively provides prospective residents with a view of the program culture. A 2008 study^9^ noted that a growing number of medical students (69%) had a Facebook account. Further, a 2016 meta-analysis by Guraya^10^ found that 70-80% of medical students utilized social networking accounts. The high social network utilization rate among medical students makes it an excellent resource to utilize for recruitment efforts. Articles such as “Twelve Tips for Utilizing Residency Program Social Media Accounts for Modified Residency Recruitment” by Bhayani et al.,^11^ offer advice on properly engaging with applicants through social media. A key takeaway is that it is vital for programs to engage in a multi-platform approach while portraying a consistent brand identity to set themselves apart from other programs.

Teleconferencing offers a final electronic resource for engaging with residents and furthering the program brand image. Many programs throughout the 2020-2021 application season offered virtual “meet and greet” events with residents and faculty. These online meetings give applicants the chance to experience the culture more personally and have their specific questions answered. Some programs even allowed applicants to view educational conferences. These events give applicants a window into the interactions between faculty and residents, furthering their brand image, and helping applicants imagine themselves at the program.

These modalities -- websites, social media, and teleconferencing -- are valuable to the resident recruitment process. Each offers different components in the recruitment toolbox, and when employed appropriately, can bolster the image that programs project and, concomitantly, the size and quality of their applicant pool. One element of paramount importance in an online strategy is the creation of a consistent brand identity that is true to the program and helps applicants to understand its culture and unique features.
